# Right Atrial Thrombus or Chiari Network?

**DOI:** 10.5811/cpcem.2017.2.32820

**Published:** 2017-05-09

**Authors:** Peter Fredericks, Theresa Liu, Joseph Colla

**Affiliations:** * University of Illinois-College of Medicine, Chicago, Illinois; † University of Illinois at Chicago, Department of Emergency Medicine, Chicago, Illinois

## CASE REPORT

A 31-year-old African-American male with known sickle cell disease presented to the emergency department (ED) with a one-week history of chest pain and bilateral leg pain. Physical examination showed an afebrile and hemodynamically stable but uncomfortable appearing male. Cardiac and respiratory exam were unremarkable. A bedside focused cardiac ultrasound (FOCUS) exam performed by the attending emergency physician (EP) revealed four dilated chambers and a hyperechoic mobile body in the right atrium (Image). The FOCUS images were forwarded to a senior cardiology fellow who confirmed that the hyperechoic body was a Chiari network and not a right atrial clot. The patient was admitted for a sickle cell vaso-occlusive crisis that was managed with morphine and intravenous fluids.

CPC-EM Capsule

What do we already know about this clinical entity?

Chiari’s network is a collection of reticula found in the right atrium that has been associated with some cardiac pathologies such as arrhythmia, paradoxical emboli, and thrombi formation.

What is the major impact of image(s)?

The major impact of this image is to inform providers of this disease entity, emphasize its defining characteristics, and highlight the associated pathologies.

How might this improve emergency medicine practice?

A Chiari’s network can prove a diagnostic challenge for emergency physicians, at times mimicking a right atrial mass, thrombus, or vegetation which may in turn lead to mistreatment.

## DISCUSSION

Found in 2% of the population, Chiari network is a collection of reticula in the right atrium that results from incomplete resorption of the Eustachian valve.^1^ The network is visualized on echocardiogram as a pulsating network of threads and fibers attached to the posterior wall of the right atrium or atrial septum. This sonographic appearance can prove to be a diagnostic challenge for EPs using point-of-care ultrasound because it could be mistaken for a right atrial mass, thrombus, or vegetation instead of an embryological remnant, which may in turn lead to mistreatment.^2^ Key elements noted on echocardiography that distinguish Chiari network include identification of at least two normal-appearing tricuspid valve leaflets and the presence of a rotary, highly mobile target that does not move into the right ventricular outflow tract or the right ventricle during diastole. While typically considered a benign anatomical variant, it has been associated with cardiac pathologies such as arrhythmia, paradoxical emboli, persistent patent foramen ovale, formation of an atrial septal aneurysm, thrombi formation, and entrapment of thrombi or catheters.^1^,^3^,^4^ Differentiating Chiari network from a more acutely pathological process is critical in the evaluation and management of hypercoagulable patients.

## Supplementary Information

Video 1 An apical four chamber view displaying a prominent Chiari network within the right atrium.

Video 2 An apical four chambier view displaying a right atrial thrombus to contrast with the Chiari network found in Video 1.

## Figures and Tables

**Image:**
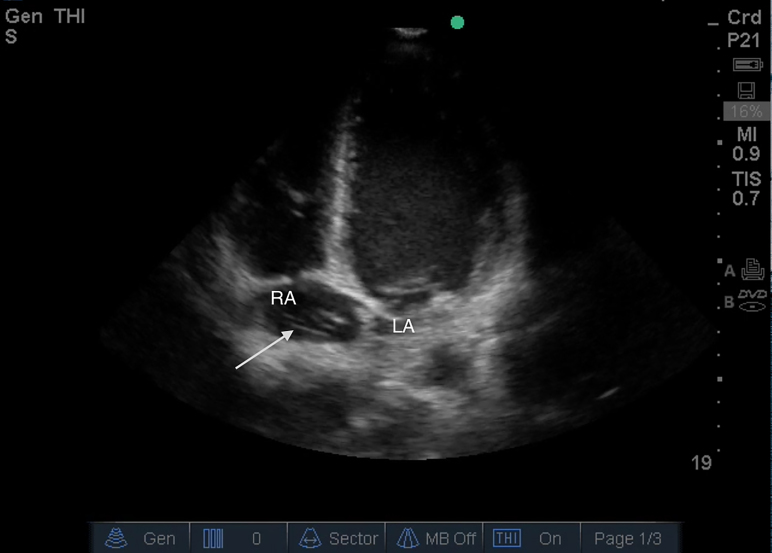
Apical four chamber view demonstrating a right atrial Chiari Network (arrow). Right Atrial Thrombus or Chiari Network? *Fredericks et al.*
